# Prevalence of disrespect and abuse of women during child birth and associated factors in Bahir Dar town, Ethiopia

**DOI:** 10.4178/epih.e2018029

**Published:** 2018-07-01

**Authors:** Biresaw Wassihun, Leul Deribe, Nadia Worede, Teklemariam Gultie

**Affiliations:** 1 College of Medicine and Health Sciences, Arba Minch University, Arba Minch, Ethiopia; 2 Department of Nursing and Midwifery, College of Health Sciences Addis Ababa University, Addis Ababa, Ethiopia

**Keywords:** Abuse, Maternity, Childbirth, Women, Prevalence, Ethiopia

## Abstract

**OBJECTIVES:**

Disrespect and abuse are an often-unacknowledged cause of maternal mortality and morbidity globally. The objective of this study was to assess the prevalence and associated factors of disrespect and abuse of women during childbirth at a health facility in the town of Bahir Dar, Ethiopia.

**METHODS:**

In this community-based cross-sectional study, 422 mothers were interviewed from March 1 to 30, 2017 using a systematic random sampling technique with the kth value of 23 calculated based on the number of households in each sub-city and the expected sample size from sub-cities. Data were collected using a structured face-to-face interview questionnaire. EpiData version 3.1 was used to code and enter data, which were analyzed using SPSS version 22. Descriptive statistics were calculated for each variable, and binary logistic regression analysis with 95% confidence intervals (CIs) was carried out to determine the associations between predictor variables and outcome variables.

**RESULTS:**

A total of 410 women participated in the study, with a response rate of 97.2%. The overall prevalence of disrespect and abuse was 67.1% (95% CI, 63.0 to 72.0). Disrespect and abuse were more prevalent in women with a monthly income less than 2,000 Ethiopian birr (adjusted odds ratio [aOR], 1.74; 95% CI, 1.08 to 2.80), mothers who stayed in a health facility after delivery (aOR, 5.14; 95% CI, 2.23 to 11.82), those who received care at a governmental hospital (aOR, 2.49; 95% CI, 1.15 to 5.40), and those who attended fewer than 4 antenatal care visits (aOR, 1.97; 95% CI, 1.15 to 3.40).

**CONCLUSIONS:**

The prevalence of disrespect and abuse was high in this study setting. To decrease the prevalence of this phenomenon, appropriate interventions should be designed, focusing on increasing the number of antenatal care visits, increasing the incomes of mothers, and improving the relationship between health workers and mothers during mothers’ stay at health facilities.

## INTRODUCTION

Disrespect and abuse are defined as any form of inhumane treatment or uncaring behavior toward a woman during labor and delivery [1]. Laboring mothers may face various forms of disrespectful and abusive treatment during childbirth at a facility, including physical abuse, lack of consent for care, non-confidential care, undignified care, abandonment, discrimination, and detention in facilities for failure to pay user fees [2].

Disrespect and abuse of women during delivery at a health facility occur globally [3]. This phenomenon has been reported to be a cause of maternal mortality and morbidity. It is a major problem that affects women during labor and delivery, and is one of the most important barriers to maternal health service utilization [4]. However, it has received less attention than other barriers to access and choice of maternal care during labor and delivery [5].

In developing countries, the lack of compassionate and respectful care during childbirth continues to raise problems, as shown by maternal mortality and morbidity that could be attributed to low maternity quality of care [6].

A study conducted in Tanzania showed that the prevalence of disrespect and abuse during childbirth was found to be 19.5% in exit interviews and 28.2% in follow-up interviews [2]. Another study conducted in Tanzania explored the prevalence of disrespect and abuse during childbirth at a health facility, and revealed that 15.0% of women reported disrespect and abuse [7].

A cross-sectional study done in southeastern Nigeria showed that 98.0% of women reported disrespect and abuse during childbirth [8]. A study conducted in Kenya indicated that 20.0% of laboring mothers experienced disrespect and abuse [9]. Furthermore, a study conducted in Addis Ababa showed that 78.0% of mothers reported disrespect and abuse [10].

The Ethiopian government has invested considerable efforts in increasing the number of health facilities and better connecting communities to facilities to improve access to and uptake of maternity services. However, recent work has suggested that improving access is not enough to increase use, and that poor perceived quality of care and poor interpersonal care discourage women from seeking delivery services at health facilities with skilled personnel [11]. Therefore, the personal interactions between clients and providers are important in shaping women’s experiences and their perceptions of maternity care during childbirth; in the long run, positive personal interactions reduce maternal mortality and morbidity [12].

Even though disrespect and abuse are an essential determinant of maternity service quality and utilization, these behaviors have received less attention than other barriers to the access and choice of maternal care during labor and delivery [1,13].

The provision of compassionate and respectful maternity care during labor and delivery promotes delivery at health facilities. Assessing the respectfulness of maternal care during childbirth is a currently core component of improving the quality of maternity services. Therefore, it is important to assess the existing prevalence of disrespect and abuse during childbirth [1,8].

However, few facility-based studies have been conducted in Ethiopia [10] and no community-based studies have been conducted on the prevalence of disrespect and abuse during childbirth and factors contributing to this behavior. Institution-based studies alone are insufficient for identifying contributing factors that are correlated with disrespect and abuse during childbirth. Thus, the objective of this study was to assess the prevalence and associated factors of disrespect and abuse during childbirth at a health facility in the town of Bahir Dar, Amhara Region, northwest Ethiopia, in 2017.

## MATERIALS AND METHODS

### Study design and setting

This community-based cross-sectional study was conducted among mothers who had given birth at a health facility within the last year in the town of Bahir Dar, Amhara Region. The study was conducted from March 1 to 30, 2017. Bahir Dar is located in the northwestern part of Ethiopia, in Amhara National Regional State, at a distance of 565 km from Addis Ababa. The total population of the town is 290,437, of whom 142,068 are men and 148,369 are women. The Bahir Dar city administration contains 9 sub-cities. In the town, there are 10 public health centers, as well as 2 public hospitals and 2 private health institutions that provide delivery services.

### Sample size determination and sampling technique

The single population proportion formula was used to calculate the sample size by hypothesizing that the proportion of mothers who were disrespected and abused during institutional delivery would be 50%, adding a non-response rate of 10%, and using the assumptions of a 95% confidence level and a 5% margin of error. The resulting sample size was 422 mothers.

Each household was selected using systematic random sampling from 9 urban sub-cities of the Bahir Dar city administration. The number of households in each sub-city was determined proportionally to the population size. To select the first household, the data collectors used the administrative office of the kebele (the smallest administrative unit in the country) and the local church as a starting point. After the first household was selected using the lottery method and the direction was determined using spinning techniques, every 23rd household was visited until the desired sample was achieved. For a household in which more than 1 mother had given birth within the last year, only 1 person was selected using the lottery method. If no one answered at a selected household during data collection, data collectors revisited the household 3 times at different time intervals, after which the household was considered as a non-respondent if the interviewers failed to conduct an interview with a mother.

### Data collection

Data were collected by face-to-face interviews using a structured questionnaire. The questionnaire was prepared in English and translated into the local language (Amharic), and then translated back into English by a different person to check the consistency. A pre-test was done with 10% of the sample size in the town of Injbara, the results of which were not included in this study. Six diploma-holding women midwives were selected to collect the data, and three Bachelor of Science-holding nurses from an area outside of the study site participated as supervisors. The data collectors and supervisors received a daylong training session on the objectives and benefits of the study, individuals’ rights, informed consent, and interview techniques. Data consistency and completeness were reviewed on a daily basis.

### Measurements

Disrespect and abuse during childbirth were measured using 7 performance standards (categories of disrespect and abuse) and their respective verification criteria developed by the Maternal and Child Health Integrated Program [14]. A total of 25 verification criteria of disrespect and abuse were used. The structured and pre-tested questionnaire was used to collect data from the study participants. The tool consisted of 3 sections. The first section was used to assess the socio-demographic characteristics of the mother. The second section was used to assess the obstetric characteristics of the participants. The third section was used to assess 7 categories of disrespect and abuse that women experienced during childbirth at a health facility (physical abuse, non‐ confidential care, non-consented care, undignified care [including verbal abuse], discrimination, abandonment or denial of care, and detention in facilities). A woman who reported at least 1 incident corresponding to the criteria asked about in a given category was considered to have experienced disrespect and abuse in the respective category [5]. If a mother was identified as having faced disrespect and abuse in at least 1 of the 7 categories, she was considered to have been disrespected and abused.

### Data analysis and interpretation

The collected data were checked manually for completion and any incomplete or misfiled questions, cleaned and stored for consistency, entered into EpiData version 3.1 (EpiData Association, Odense, Denmark), and then exported to SPSS version 22.0 (IBM Corp., Armonk, NY, USA) for analysis. Descriptive statistics were calculated and presented using tables and figures. Multivariable logistic regression analysis was performed to adjust for possible confounding variables. Variables that were significant in the bivariate logistic regression were entered into the multiple regression analysis. The p< 0.05 or 95% confidence intervals (CIs) not including 1.0 were considered to indicate statistical significance.

### Ethical considerations

Ethical clearance and approval were obtained from the ethics committee of the Department of Nursing and Midwifery, College of Health Science, Addis Ababa University. A letter from the research ethics committee was then submitted to the Bahir Dar Regional Health Bureau and the selected sub-cities. After explaining the objectives of the study in detail, informed verbal consent was obtained from all study participants. All the participants were reassured of anonymity, as no personal identifiers were used. Then, after obtaining informed consent from every participant, the data collectors continued to show due respect to the norms, values, beliefs, and culture of the participants, and the confidentiality of the data was ensured.

## RESULTS

### Socio-demographic characteristics of the study population

Of the 422 mothers who were invited for an interview, 410 participated in the study, giving a response rate of 97.2%. The mean age of the respondents was 28.6 years, with a standard deviation of 4.6 years and minimum and maximum ages of 16.0 and 48.0 years, respectively. The plurality of the respondents (n= 184, 45.0%) were aged 25-29 years. The majority (n=366, 89.3%) of the study participants were from the Amhara ethnic group and 327 (79.7%) were Orthodox Christian. Regarding the marital status of the mother, 348 (84.9%) were married, and 226 (55.1%) had a monthly family income less than 2,000 Ethiopian birr (ETB). In addition, almost all the respondents (n= 346, 84.4%) did not have the financial capacity to pay for delivery services (Table 1).

### Obstetric history of the study participants

Of the respondents, 404 (98.5%) had a history of antenatal care (ANC) service utilization for their most recent delivery. Around half (44.9%) of the mothers received care from a midwife for ANC services. The majority (87.3%) of mothers who received ANC services attended government health centers. Two hundred seventy nine (68.0%) of the respondents attended greater than or equal to 4 ANC visits, and 178 participants (43.4%) had a previous history of institutional delivery at a government hospital only once (Table 2).

### Prevalence of disrespect and abuse during childbirth at a health facility

Of the 410 respondents who were interviewed, 275 (67.1%) reported having experienced at least 1 form of disrespect and abuse during childbirth at a health facility, while only 135 (32.9%) did not experience any form of disrespect and abuse.

### Type of disrespect and abuse during childbirth at a health facility

**Figure 1.**
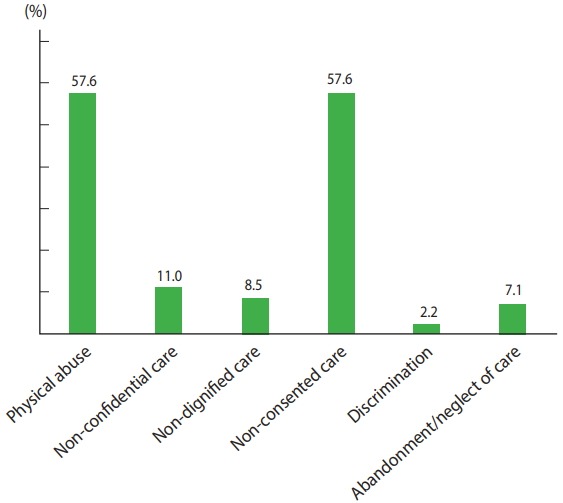
Frequency of disrespect and abuse by category during facility based childbirth in Bahir Dar town, North West, Ethiopia, March 1 to 30, 2017 (n= 410). Values are presented as percentage each from 100%.

Of the types of abuse and disrespect that were studied, physical abuse (n= 236, 57.6%) and non-consented care (n= 236, 57.6%) were the most prevalent types, followed by non-confidential care (n=45, 11.0%) (Figure 1). The most commonly experienced form of physical abuse was lack of care in a culturally appropriate way (n= 193, 47.1%), followed by being verbally insulted by the provider (n=111, 27.1%). The commonly reported criterion in the domain of non-consented care was ‘provider did not allow me to assume my position of choice during birth’ (n= 154, 37.6%) followed by ‘provider did not introduce himself/herself to me and my companion’ (n= 126, 30.7%). From the domain of non-confidential care, 34 (8.3%) of women reported that healthcare providers did not use drapes or covering appropriate for protecting the mother’s privacy. Detention in the health facility was the least commonly reported (n= 5, 1.2%) form of abuse and disrespect that was studied (Table 3).

### Factors associated with disrespect and abuse during childbirth

Binary logistic regression was performed to assess the association of each independent variable with disrespect and abuse. Factors that showed a p-value of 0.2 or less were included in the multivariable regression model. The results of the bivariate analysis showed that the monthly family income of the respondent, the number of ANC visits, gravidity, stay at a health facility after delivery, mode of delivery, complications during delivery, and the sex of the healthcare provider attending the delivery were significantly associated with disrespect and abuse.

The result of multiple logistic regression analysis showed that, the monthly family income of the respondent, the number of ANC visits, the type of health facility, and the length of stay at a health facility were significantly associated with disrespect and abuse (p<0.05). Respondents with a monthly family income of < 2,000 ETB were 1.74 times more likely to have been disrespected and abused than those who had a family monthly income of ≥ 2,000 ETB (adjusted odds ratio [aOR], 1.74; 95% CI, 1.08 to 2.80; p=0.02). Respondents with a history of fewer than 4 ANC visits were 1.97 times more likely to have been disrespected and abused than respondents with a history of ≥4 ANC visits (aOR, 1.97; 95% CI, 1.15 to 3.40; p=0.01). Similarly, respondents who gave birth at a governmental hospital were 2.49 times more likely to have been disrespected and abused than mothers who gave birth at a private health institution (aOR, 2.49; 95% CI, 1.15 to 5.40; p=0.02). In addition, mothers who stayed longer at a health facility after delivery were 5.14 times more likely to have been disrespected and abused than those who did not (aOR, 5.14; 95% CI, 2.23 to 11.82; p= 0.01) (Table 4).

## DISCUSSION

This study identified the prevalence and associated factors of disrespect and abuse of women during childbirth at a health facility. In this study, two out of three women reported disrespect and abuse during childbirth. This proportion is slightly lower than was reported in a previous study conducted in Addis Ababa, which showed that the prevalence of disrespect and abuse was 78.0% [9]. This discrepancy might be due to the small sample size of the previous study and differences in the measurement tool that was used.

In contrast, this rate is higher than was reported in a study conducted in Tanzania and Kenya, which showed 15.0 and 20.0% prevalence, respectively [9]. This discrepancy may be due to sociocultural and socioeconomic differences that affect professionals’ behavior and their reactions in the context of clinical care. Even though the Ethiopian Ministry of Health advocates for companionate and respectful care in all settings, this finding indicates that there is a greater need to improve the maternity care that women receive.

The most frequently reported forms of disrespect and abuse were physical abuse and non-consented care, which were also found to be common in a Nigerian study [8]. Women are often not given a chance to select the type of care they want to receive, and very minimal information on the childbirth process is given. Asking women for consent is an important measure of showing respect for the laboring mother. In this study, three of five laboring mothers received non-consented care, which is a higher rate than was reported in studies conducted in Tanzania (3.5%) [2], Ghana (35.7%) [8], and Kenya (4.2%) [9].

During childbirth, physical abuse can occur in various forms, ranging from caring in a culturally inappropriate way to insulting or even hitting. In low-income countries where delivery at a health institution is less common, this kind of behavior on the part of health care providers will further decrease women’s intention to give birth at health facilities. It is also women’s right to receive respectful maternity care. This study revealed that a significant number of mothers had received care in a culturally inappropriate way (47.1%), been subjected to physical force or slapped (23.2%), and insulted (27.1%) by healthcare providers.

The declaration of the universal rights of childbearing women states that healthcare providers must protect the patient’s privacy and confidentiality during any procedure and when handling a woman’s information [15]. In contrast, this study revealed that 11.0% of women had been provided care in a non-confidential manner. This could be due to the lack of appropriate physical barriers at health facilities and/or poor understanding of the importance of confidentiality during childbirth among healthcare providers. This rate is lower than was reported in a study in Malawi [16] and higher than was reported in a study in urban Tanzania (2.0%) [7] and Kenya (8.5%) [9]. This inconsistency might be due to differences in healthcare policies and programs that have been implemented.

According to our findings, the next most common category of disrespect and abuse experienced by women was non-dignified care (8.5%). This rate is lower than was reported in studies conducted in Ghana (29.6%) and Kenya (18.0%) [9,17]. This discrepancy might be due to differences in the study period and location.

Discrimination during the provision of service was reported by 2.2% of the participants in this study. The final category of disrespect and abuse reported in this study was abandonment/neglectful care during labor and delivery, which was reported by 7.1% of participants. This rate is lower than was observed in a direct observational study conducted in 5 countries, including Ethiopia, Kenya, Madagascar, Nigeria, and Tanzania (range, 9 to 29%) [18]. This inconsistency may be due to the small sample size of this study and differences in the study period.

In this study, maternal family income was significantly associated with disrespect and abuse. Mothers who had a low family income were more likely to have experienced disrespect and abuse. This finding is consistent with a similar study that was conducted in Addis Ababa, Ethiopia [10]. This result is also similar to that of a study conducted in Bangladesh and Kenya, which showed that affluent women received care earlier than their poor counterparts, regardless of the seriousness of their medical condition [13,16].

The findings of this study also showed that as the number of ANC visits increased, women were less likely to be disrespected. In addition, reports of disrespect and abuse increased with the length of a woman’s stay at a health facility. This finding is consistent with a study conducted in Tanzania [2].

In conclusion, this study revealed that the prevalence of disrespect and abuse during labor and delivery was high (67.1%) in the town of Bahir Dar, Ethiopia.

The majority of mothers who gave birth, especially at governmental health institutions, experienced at least 1 form of disrespect and abuse. Monthly family income, the number of ANC visits, the type of health facility, and the length of stay at a health facility after delivery were factors significantly associated with disrespect and abuse.

Abusive and disrespectful care at health facilities is a serious concern, which merits due attention to promote women-friendly care for all women. It is essential that the economic empowerment of women and increasing the number of ANC visits be prioritized in order to improve access to high-quality medical services and to minimize disrespect and abusive care.

## Figures and Tables

**Table 1.** Socio-demographic characteristics of mothers in the town of Bahir Dar, northwest Ethiopia, March 1 to 30, 2017 (n=410)

Variables	Frequency (%)
Age (yr)	
15-19	6 (1.5)
20-24	58 (14.1)
25-29	184 (44.9)
30-34	108 (26.3)
≥35	54 (13.2)
Marital status	
Single	35 (8.5)
Married	348 (84.9)
Divorced	21 (5.1)
Widowed	6 (1.5)
Religion	
Orthodox	327 (79.7)
Muslim	67 (16.3)
Others^1^	16 (3.9)
Ethnicity	
Amhara	366 (89.3)
Agew	24 (5.8)
Others^2^	20 (4.9)
Educational background	
No formal education	105 (25.6)
Primary	71 (17.3)
Secondary	124 (30.2)
College and above	110 (26.8)
Occupation	
Housewife	196 (47.8)
Private employee	54 (13.2)
Government employee	75 (18.3)
Merchant	69 (16.8)
Other^3^	16 (3.9)
Family monthly income (ETB)	
≥2,000	226 (55.1)
<2,000	184 (44.9)
Ability to pay for delivery services	
Yes	64 (15.6)
No	346 (84.4)

ETB, Ethiopian birr.

1 Protestant and Catholic.

2 Oromo, Tigray, Sidama, Gurage, and Wolayita.

3 Daily laborers and students.

**Table 2.** Obstetric characteristics of mothers in the town of Bahir Dar, northwest Ethiopia, March 1 to 30, 2017 (n=410)

Characteristics	Frequency (%)
ANC	
Yes	404 (98.5)
No	6 (1.5)
Place of ANC	
Government health facility	358 (87.3)
Private health facility	52 (12.7)
Care received from	
Doctors	86 (21.0)
Nurses or midwifes	324 (79.0)
No. of ANC visits	
<4	131 (31.9)
≥4	279 (68.0)
Gravidity	
1	135 (32.9)
2-3	160 (39.0)
≥4	115 (28.0)
No. of deliveries at health facilities	
All	78 (19.0)
1	178 (43.4)
3	45 (11.0)
4	16 (3.9)
No. of days stayed at the health facility after delivery	
<3	272 (66.3)
≥3	138 (33.7)

ANC, antenatal care.

**Table 3.** Category and type of disrespect and abuse reported by mothers during childbirth, Bahir Dar, 2017

Categories	Types of disrespect and abuse	Yes	No
Physical abuse	The provider used physical force/slapped me/hit me	95 (23.2)	315 (76.8)
	The provider verbally insulted me during labor	111 (27.1)	299 (72.9)
	I was separated from my baby without medical indication	13 (3.2)	397 (96.8)
	Support staff insulted me and my companion	15 (3.6)	395 (96.3)
	The providers did not demonstrate caring in a culturally appropriate way	193 (47.1)	217 (52.9)
	Received unnecessary pain-relief treatment	36 (8.8)	374 (91.2)
	Denied food or fluids in labor unless medically necessitated	7 (1.7)	403 (98.3)
Non-confidential care	The provider did not use curtains or other visual barriers for protecting privacy	34 (8.3)	376 (91.7)
	Providers discussed my private health information in a way that others could hear	22 (5.4)	388 (94.6)
Non-consented care	The provider did not introduce himself/herself to me and my companion	126 (30.7)	284 (69.3)
	The provider did not encourage me to ask questions	99 (24.1)	311(75.8)
	The provider did not respond to my questions with promptness, politeness, and truthfulness	82 (20.0)	328 (80.0)
	The provider did not explain to me what was being done and what to expect throughout labor and birth	112 (27.3)	298 (72.7)
	The provider did not give me periodic updates on status and progress of my labor	70 (17.1)	340 (82.9)
	The provider did not allow me to assume my position of choice during birth	154 (37.6)	256 (62.4)
	The provider did not obtain my consent or permission prior to any procedure	103 (25.1)	307 (74.9)
Non-dignified care	Providers shouted at or scolded me during labor	32 (7.8)	378 (92.2)
	Providers made negative comments during labor	10 (2.4)	400 (97.6)
Abandonment/neglect of care	The provider left me alone or unattended	8 (1.9)	402 (98.0)
	The provider did not come quickly when I called him/her	26 (6.3)	384 (93.6)
Discrimination	Healthcare providers discriminated by race, ethnicity, or economic status	8 (1.9)	402 (98.0)
	Healthcare providers discriminated because of being a teenager	1 (0.2)	409 (99.7)
	Healthcare providers discriminated because of being HIV-positive	3 (0.7)	407 (99.3)
Detention in a health facility	Discharge was postponed until hospital bills were paid	0 (0.0)	410 (100.0)
	I was detained in a health facility against my will	5 (1.2)	405 (98.8)

Values are presented as number (%). HIV, human immunodeficiency virus.

**Table 4.** Factors associated with disrespect and abuse during labor and delivery among mother in the town of Bahir Dar, northwest Ethiopia, March 1 to 30, 2017 (n= 410)

Variables	Experience of disrespect and abuse		OR (95% CI)	aOR (95% CI)
	Yes	No		
Age (yr)				
15-24	45 (71.4)	18 (28.6)	1.00 (reference)	1.00 (reference)
25-34	197 (67.5)	95 (32.5)	0.83 (0.46, 1.51)	1.05 (0.52, 2.12)
≥35	33 (60.0)	22 (40.0)	0.60 (0.28, 1.29)	0.40 (0.15, 1.07)
Educational status				
No formal education	71 (67.6)	34 (32.4)	1.13 (0.69, 1.84)	0.88 (0.50, 1.56)
Primary	52 (73.2)	19 (26.8)	1.48 (0.82, 2.66)	1.10 (0.57, 2.15)
Secondary and above	152 (65.0)	82 (35.0)	1.00 (reference)	1.00 (reference)
Monthly income (ETB)				
<2,000	166 (73.5)	60 (26.5)	1.90 (1.25, 2.88)^*^	1.74 (1.08, 2.80)^*^
≥2,000	109 (59.2)	75 (40.8)	1.00 (reference)	1.00 (reference)
No. of antenatal care visits				
<4	104 (79.4)	27 (20.6)	2.43 (1.49, 3.96)^*^	1.97 (1.15, 3.40)^*^
≥4	171 (61.3)	108 (38.7)	1.00 (reference)	1.00 (reference)
Gravidity				
1	92 (68.1)	43 (31.9)	1.00 (reference)	1.00 (reference)
2-3	91 (56.9)	69 (43.1)	0.62 (0.38, 0.99)^*^	0.73 (0.42, 1.27)
≥4	92 (80.0)	23 (20.0)	1.87 (1.04, 3.35)^*^	1.64 (0.81, 3.33)
Types of health facility				
Government health facility	249 (68.6)	114 (31.4)	1.76 (0.95, 3.26)	2.49 (1.15, 5.40)^*^
Private health facility	26 (55.3)	21 (44.7)	1.00 (reference)	1.00 (reference)
Mode of delivery				
Spontaneous vaginal	144 (58.8)	101 (41.2)	2.70 (1.70, 4.26)^*^	1.20 (0.66, 2.22)
Instrumental	131 (79.4)	34 (20.6)	1.00 (reference)	1.00 (reference)
Gender				
Men	148 (72.2)	57(27.8)	1.59 (1.05, 2.41)^*^	1.43 (0.87, 2.33)
Women	78 (38.0)	127 (62.0)	1.00 (reference)	1.00 (reference)
Complications during delivery				
Yes	96 (88.1)	13 (11.9)	5.03 (2.69, 9.38)^*^	1.49 (0.60, 3.70)
No	179 (59.5)	122 (40.5)	1.00 (reference)	1.00 (reference)
Long stay at the health facility				
Yes	124 (89.9)	14 (10.1)	7.10 (3.88, 12.9)^*^	5.14 (2.23, 11.82)^*^
No	151 (55.5)	121 (44.5)	1.00 (reference)	1.00 (reference)

Values are presented as frequency (%). OR, odds ratio; CI, confidence interval; aOR, adjusted OR; ANC, antenatal care; ETB, Ethiopian birr.

* p <0.05.

## References

[1] Bowser D, Hill K (2010). Exploring evidence for disrespect and abuse in facility-based childbirth. Report of a landscape analysis. https://www.ghdonline.org/uploads/Respectful_Care_at_Birth_9-20-101_Final1.pdf.

[2] Kruk ME, Kujawski S, Mbaruku G, Ramsey K, Moyo W, Freedman LP (2018). Disrespectful and abusive treatment during facility delivery in Tanzania: a facility and community survey. Health Policy Plan.

[3] Freedman LP, Kruk ME (2014). Disrespect and abuse of women in childbirth: challenging the global quality and accountability agendas. Lancet.

[4] World Health Organization (2015). Health in 2015: from MDGs to SDGs. http://www.who.int/gho/publications/mdgs-sdgs/en/.

[5] World Health Organization (2014). Trends in maternal mortality: 1990 to 2013: estimates by WHO, UNICEF,UNFPA, the World Bank and the United Nations Population Division. http://www.who.int/reproductivehealth/publications/monitoring/maternal-mortality-2013/en/.

[6] Hulton LA, Matthews Z, Stones RW (2000). A framework for the evaluation of quality of care in maternity services. https://eprints.soton.ac.uk/40965/1/12757_Matthews.pdf.

[7] Sando D, Kendall T, Lyatuu G, Ratcliffe H, McDonald K, Mwanyika-Sando M (2014). Disrespect and abuse during childbirth in Tanzania: are women living with HIV more vulnerable?. J Acquir Immune Defic Syndr.

[8] Okafor II, Ugwu EO, Obi SN (2015). Disrespect and abuse during facility-based childbirth in a low-income country. Int J Gynaecol Obstet.

[9] Abuya T, Warren CE, Miller N, Njuki R, Ndwiga C, Maranga A (2015). Exploring the prevalence of disrespect and abuse during childbirth in Kenya. PLoS One.

[10] Asefa A, Bekele D (2015). Status of respectful and non-abusive care during facility-based childbirth in a hospital and health centers in Addis Ababa, Ethiopia. Reprod Health.

[11] Koblinsky M, Tain F, Tesfaye S (2010). Reducing maternal mortality and increasing use of skilled birth attendance: Ethiopia and MDG 5. Ethiop J Reprod Health.

[12] Bohren MA, Hunter EC, Munthe-Kaas HM, Souza JP, Vogel JP, Gülmezoglu AM (2014). Facilitators and barriers to facility-based delivery in low- and middle-income countries: a qualitative evidence synthesis. Reprod Health.

[13] Warren C, Njuki R, Abuya T, Ndwiga C, Maingi G, Serwanga J (2013). Study protocol for promoting respectful maternity care initiative to assess, measure and design interventions to reduce disrespect and abuse during childbirth in Kenya. BMC Pregnancy Childbirth.

[14] Reis V, Deller B, Carr CC, Smith J (2012). Respectful maternity care. https://www.k4health.org/sites/default/files/RMC%20Survey%20Report_0.pdf.

[15] Windau-Melmer T (2013). A guide for advocating for respectful maternity care. https://www.healthpolicyproject.com/pubs/189_RMCGuideFINAL.pdf.

[16] Sethi R, Gupta S, Oseni L, Mtimuni A, Rashidi T, Kachale F (2017). The prevalence of disrespect and abuse during facility-based maternity care in Malawi: evidence from direct observations of labor and delivery. Reprod Health.

[17] Moyer CA, Adongo PB, Aborigo RA, Hodgson A, Engmann CM (2014). ‘They treat you like you are not a human being’: maltreatment during labour and delivery in rural northern Ghana. Midwifery.

[18] Rosen HE, Lynam PF, Carr C, Reis V, Ricca J, Bazant ES (2015). Direct observation of respectful maternity care in five countries: a cross-sectional study of health facilities in East and Southern Africa. BMC Pregnancy Childbirth.

[19] Pitchforth E, van Teijlingen E, Graham W, Dixon-Woods M, Chowdhury M (2006). Getting women to hospital is not enough: a qualitative study of access to emergency obstetric care in Bangladesh. Qual Saf Health Care.

